# Glycemic status, non-traditional risk and left ventricular structure and function in the Jackson Heart Study

**DOI:** 10.1186/s12872-022-02605-w

**Published:** 2022-04-21

**Authors:** Chizobam Ani, David Shavlik, Synnove Knutsen, Islam Abudayyeh, Jimmie Banta, Emily O’Brien, Robert J. Mentz, Alain G. Bertoni, Gary Fraser

**Affiliations:** 1 Loma Linda University (LLU), Loma Linda, USA; 2 Department of Internal Medicine, Charles R Drew University of Medicine and Science (CDU), Los Angeles, USA; 3 University of California Los Angeles (UCLA), Los Angeles, USA; 4 Duke University, Durham, USA; 5 Wake Forest School of Medicine (Department of Epidemiology and Prevention), Winston-Salem, USA

**Keywords:** Diabetic cardiomyopathy, Left ventricular structure, Diabetes, African American

## Abstract

**Background:**

Left ventricular structure and function abnormalities may be an early marker of cardiomyopathy among African Americans with diabetes (DM) even in the absence of coronary artery disease (CAD), arrhythmia, valvular heart disease and end-stage renal disease (ESRD). This study examined the association of prediabetes (PDM), DM and HbA1c with left ventricular structure and function among Jackson Heart Study (JHS) participants without traditional risk factors.

**Methods:**

Retrospective cross-sectional analyses of the association of PDM, DM and HbA1c with, left ventricular ejection fraction (LV EF), fractional shortening (LV FS), stroke volume index (SVI), cardiac index (CI), left ventricular end diastolic volume index (LVEDVI), left ventricular end systolic volume index (LVESVI), relative wall thickness (RWT), myocardial contraction fraction (MCF) and left ventricular mass index (LVMI). The study was conducted in 2234 adult JHS participants without preexisting CAD, arrhythmia, valvular heart disease or ESRD. Statistical analyses included descriptive, univariate and covariate adjusted linear regression analyses. Sensitivity analyses to explore the impact of hypertension on study outcomes were also carried out.

**Results:**

DM compared with no DM was associated with lower, SVI (− 0.96 ml/m^2^, *p* = 0.029), LVEDVI (− 1.44 ml/m^2^ *p* = 0.015), and MCF (− 1.90% p = 0.007) but higher CI (0.14 L/min/m^2^, *p* < 0.001), RWT (0.01 cm, *p* = 0.002) and LVMI (2.29 g/m^2^, *p* = 0.009). After further control for DM duration, only CI remaining significantly higher for DM compared with no DM participants (0.12 L/min/m^2^, *p* = 0.009). PDM compared with no PDM was associated with lower, SVI (− 0.87 ml/m^2^, P = 0.024), LVEDVI (− 1.15 ml/m^2^ *p* = 0.003) and LVESVI (− 0.62 ml/m^2^ *p* = 0.025). HbA1c ≥ 8.0% compared with HbA1c < 5.7% was associated with lower SVI (− 2.09 ml/m^2^, *p* = 0.004), LVEDVI (− 2.11 ml/m^2^ *p* = 0.032) and MCF (− 2.94% *p* = 0.011) but higher CI (0.11 L/min/m^2^, *p* = 0.043) and RWT (0.01 cm, *p* = 0.035).

**Conclusions:**

Glycemic status is associated with important left ventricular structure and function changes among African Americans without prior CAD, arrhythmia, valvular heart disease and ESRD. Longitudinal studies may further elucidate these relationships.

**Supplementary Information:**

The online version contains supplementary material available at 10.1186/s12872-022-02605-w.

## Background

Type I and II diabetes mellitus (DM) affects proximately 30.3 million adults in the United States (US) and is expected to double in prevalence by 2050 [1]. Prediabetes (PDM) affects about 91.8 million adults with an annualized conversion rate to DM of 5–10% [2, 3]. DM has been reported to increase the risk of heart failure (HF) 2 to fivefold and about 19–30% of HF patients have concurrent DM [4–8]. Furthermore, glycemic status may be associated with poor HF outcomes among individuals with DM [9].

Rubler et al. proposed a unique association between DM and structural cardiac changes in the absence of major coronary artery disease (CAD) or valvular heart disease often termed diabetic cardiomyopathy (D-CM) [10]. While its pathophysiology and clinical course remains unclear, D-CM is increasingly recognized as a DM complication. Suggested pathophysiologic mechanisms for D-CM include; hyperglycemia, insulin resistance, myocardial fibrosis, small vessel disease and cardiac autonomic neuropathy [11, 12]. While convention suggests that diastolic dysfunction precedes left ventricular (LV) systolic dysfunction [12], emerging evidence suggests however that LV structural changes and systolic dysfunction may occur early, precede diastolic dysfunction and be an early marker of D-CM [13–16].

African Americans are at higher risk of DM and HF when compared with other ethnicities [17–19]. While traditional risk factors like CAD, valvular heart disease and arrythmia have been associated with HF in individuals with DM, few studies have explored the independent relationship between glycemic status and Left ventricular structure and function (LV SF) among African Americans [20].

## Methods

### Study aim

This study examined the association of PDM, DM and glycated hemoglobin (HbA1c) with LV SF among African American participants in the Jackson Heart Study (JHS) without prior (CAD), arrhythmia, valvular heart disease or end stage renal disease (ESRD).

### Design, setting and data

This was a cross sectional analysis of the JHS baseline data. JHS is a community-based cohort study that explores the risk and etiologic factors for cardiovascular disease among African Americans. JHS commenced in 2000 and includes a cohort of 5,306 participants from the Jackson, Mississippi metropolitan statistical area. Participants were selected from 4 recruitment pools: random (17%), volunteer (22%), Atherosclerosis Risk in Communities (ARIC) Study (30%), and secondary family members (31%). Study design and methods have been described previously [21].

### Characteristics of study participants (Fig. 1)

**Fig. 1:**
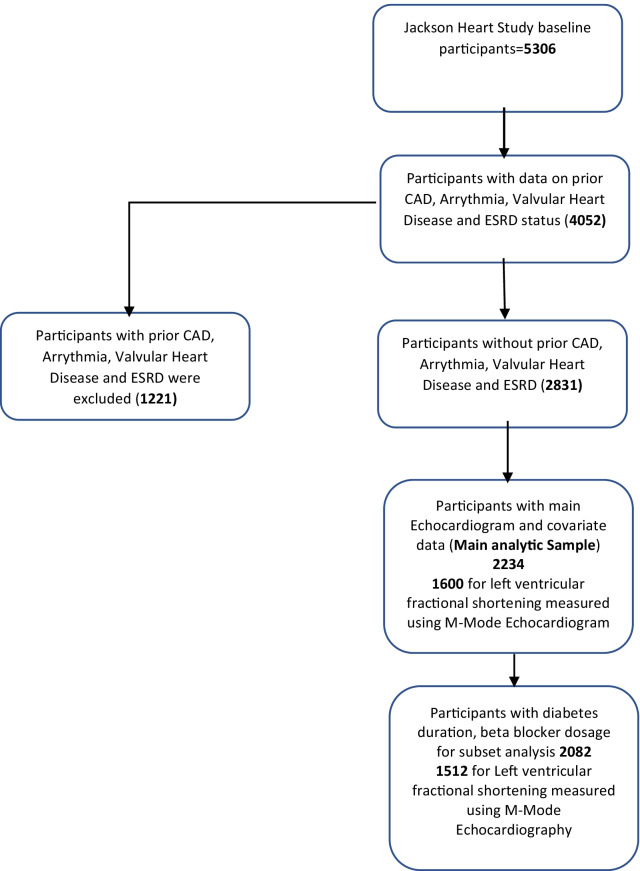
Study sample selection flow diagram

From the 5306 JHS study baseline participants, 4052 participants had complete CAD, arrythmia, valvular heart disease and ESRD data. From these, 1221 participants with prior CAD, arrhythmia, valvular heart disease and ESRD were excluded. From the 2831 participants remaining, 2234 and 1600 participants with 2D and M-Mode echocardiogram data respectively with pertinent study covariates were selected as the final analytical sample. The diabetes duration and beta blocker dosage subset analyses were conducted on 2082 and 1512 participants for the 2D echocardiogram and M-Mode outcomes respectively. Main study exclusion criteria were (a) CAD (self-report, clinical or EKG evidence of prior myocardial infarction), (b) History of significant arrhythmia (atrial flutter or fibrillations and major ventricular tachyarrhythmias) (c) valvular heart disease (moderate to severe aortic, mitral, tricuspid or pulmonary disease) and (d) ESRD on hemodialysis.

## Measures

### Outcome measures

Echocardiography was performed using Sonos 4500 echocardiogram Hewlett Packard machines following American Society of Echocardiography recommendations [22]. 2D and M-mode examination assessed all 4 cardiac chamber parasternal, apical, and subcostal windows long axis views. Blinded observers then read and provided quality ratings [23]. Nine left ventricular structure and function measures were examined; (a) Left ventricular ejection fraction % (LV EF) using biplane Simpson’s method. (b) Left ventricular end diastolic volume index (LVEDVI) = left ventricular end diastolic (LVEDV)/Body surface area (BSA) and left ventricular end systolic volume index (LVESVI) = left ventricular end systolic volume (LVESV)/BSA), (c) Stroke volume index (SVI) = LVEDV- LVESV/ BSA; (d) Cardiac index (CI) = heart rate at echocardiogram image acquisition × corresponding stroke volume)/BSA, (e) Left ventricular fractional shortening (LV FS) = Left ventricular end diastolic volume (LVEDD)—Left ventricular end systolic volume (LVEDD)/LVESD) × 100; (f) Left ventricular mass index (LVMI) = left ventricular mass (LVM) in g = [((0.8 × 1.04) (LVEDD + interventricular septal thickness + posterior wall thickness)^3^)) − (LVEDD)^3^ + 0.6/BSA], (g) Myocardial contraction fraction (MCF) = ratio of stroke volume to left ventricular myocardial volume (LVM/1.04 g/mL) [24] and (h) Relative wall thickness (RWT) was calculated as 2 × posterior wall thickness/LVEDD. All measurements utilized the 2D echocardiogram values, except for LV FS which utilized M-Mode values.

### Main independent measures

DM and PDM were the main independent variables. DM was defined by; self-reported physician diagnosis, medication use (oral or insulin) or HbA1c ≥ 6.5%. PDM was defined as HbA1c 5.7–6.4% in the absence of prior DM diagnosis or medication use. HbA1c was the secondary independent measure categorized as HbA1c < 5.7%, 5.7 to < 6.5%, 6.5% to < 8.0% and > 8.0%.

### Covariates

JHS clinic procedures are reported previously [21, 25]. Covariates include (a) Hypertension, (b) Dyslipidemia, (c) CKD stage III-IV, (d) smoking status, (e) Nutrition status (using a 158 question food frequency questionnaire and 24 h dietary recall [26] categorized using American heart association (AHA) criteria [27], (f) physical activity- similarly using AHA’s Life's Simple 7 criteria [27], (g) Socio-demographic variables (age, gender and highest level of education) (h) crack or cocaine use, (i) Alcohol use and (j) Body mass index (BMI). Cardioactive medications; obtained using JHS Medication survey form (MSRA) were considered given their potential impact on cardiac remodeling [28] and include; beta or calcium channel blockers, diuretics, angiotensin converting enzyme inhibitor (ACE) and angiotensin receptor blockers (ARB). Vasodilators were excluded from consideration given insufficient records. Beta blockers were converted to carvedilol equivalent doses for subset analyses using methods described previously by Cohen-Solal et al. [29] Finally, left ventricular hypertrophy patterns were approximated using a composite of LVMI and RWT and classified as; No LVH, concentric remodeling, eccentric hypertrophy and concentric hypertrophy [30].

## Statistical analysis

**Fig. 2:**
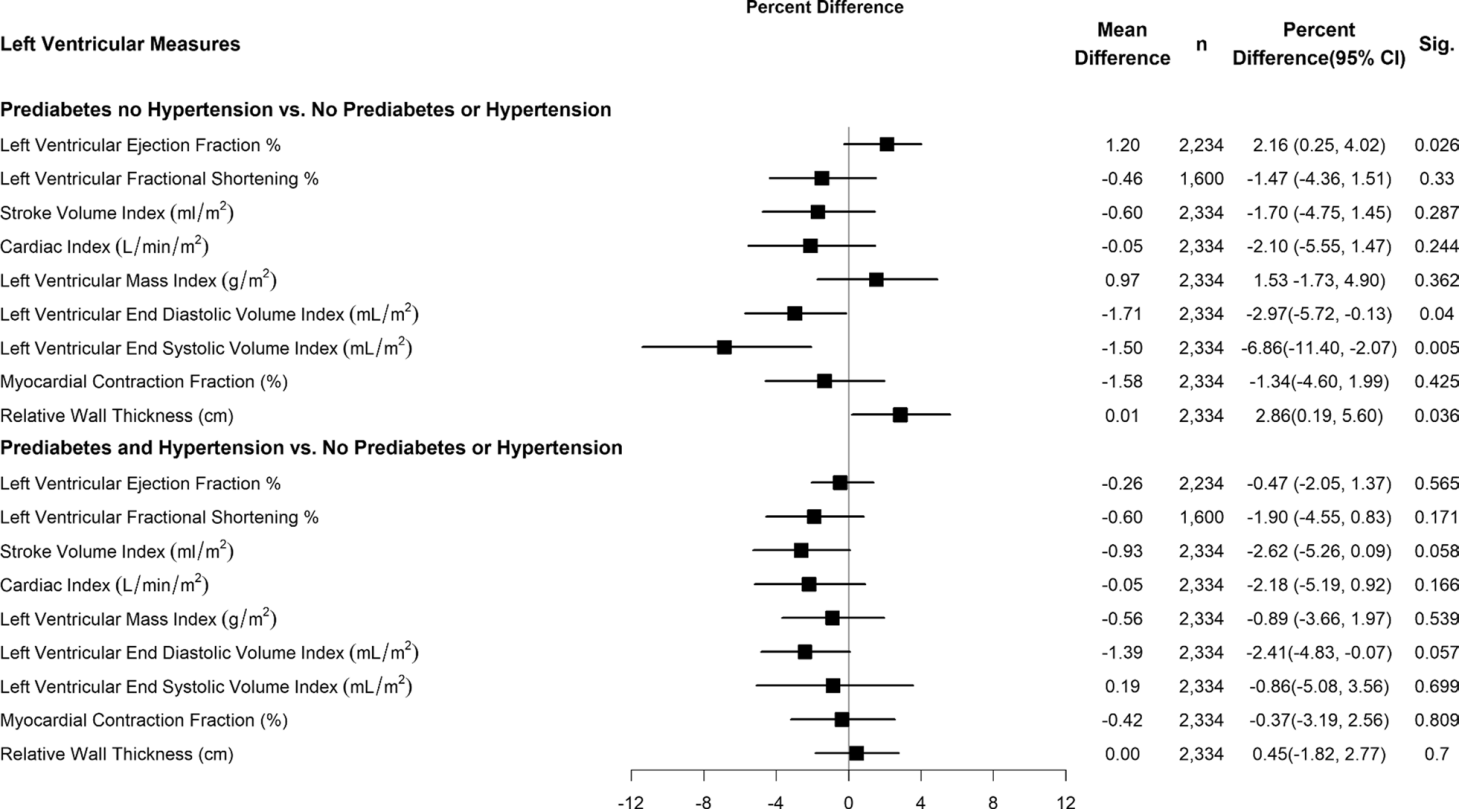
Hypertension and PDM interaction versus LV outcomes (Reference = No PDM, DM or hypertension). Each row represents a separate model for Left Ventricular Measures controlled for age gender physical activity, highest level of education, nutrition pattern, Dyslipidemia smoking status, used crack or cocaine in any form, beta blocker, Calcium channel blockers, diuretics, Angiotensin converting enzyme inhibitor, Angiotensin Receptor Blocker. LVEF, LV FS, MCF and RWT were additionally controlled for log of BMI. SVI: P for prediabetes and hypertension interaction = 0.654. CI: P for prediabetes and hypertension interaction = 0.974. LMVI: P for prediabetes and hypertension interaction = 0.270. LV EF: P for prediabetes and hypertension interaction = 0.354. LV FS: P for prediabetes and hypertension interaction = 0.830. LVEDVI: P for prediabetes and hypertension interaction = 0.767. LVESVI: P for prediabetes and hypertension interaction = 0.062. MCF: P for prediabetes and hypertension interaction = 0.652 and RWT: P for prediabetes and hypertension interaction = 0.174

**Fig. 3:**
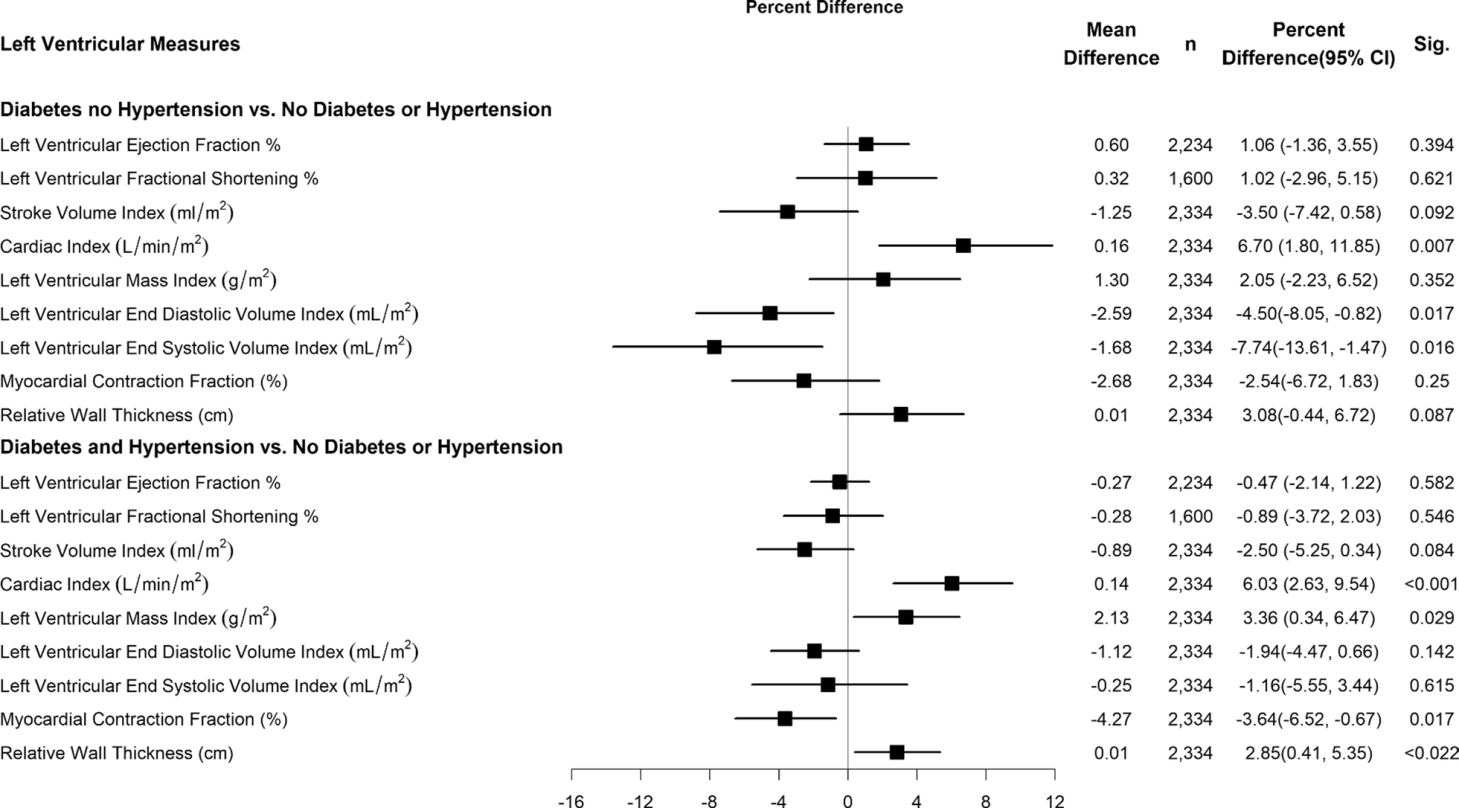
Hypertension and DM interaction versus LV outcomes (Reference = No PDM, DM or hypertension). Each row represents a separate model for Left Ventricular Measures controlled for age gender physical activity, highest level of education, nutrition pattern, Dyslipidemia smoking status, used crack or cocaine in any form, beta blocker, Calcium channel blockers, diuretics, Angiotensin converting enzyme inhibitor, Angiotensin Receptor Blocker. LVEF, LV FS, MCF and RWT were additionally controlled for log of BMI. SVI P for Diabetes and hypertension interaction = 0.680. CI: P for Diabetes and hypertension interaction = 0.824. LVMI: P for Diabetes and hypertension interaction = 0.625. LV EF:P for Diabetes and hypertension interaction = 0.292. LV FS:P for Diabetes and hypertension interaction = 0.434. LVEDVI P for Diabetes and hypertension interaction = 0.250. LVESVI P for Diabetes and hypertension interaction = 0.085. MCF: P for Diabetes and hypertension interaction = 0.666. RWT:P for Diabetes and hypertension interaction = 0.917

The distribution of all study variables was examined and positively skewed variables were transformed using their natural logarithms. Categorical variables were examined by PDM or DM status using Chi-square and Fishers exact test. Continuous measures were examined using one-way analysis of variance including Kruskal–Wallis tests when normality and homoscedasticity assumptions were not met. Simple linear regression analyses tested the univariate relationship between each LV SF outcomes with PDM or DM status (No PDM/DM, PDM and DM). Underlying linear regression assumption tests determined that all the outcome measures did not meet the normality assumptions and were therefore transformed using their natural logarithm. The coefficients of these log transformed measures were converted to percent differences using the reverse transformation formula (exponentiated (β Coefficients) – 1) × 100. Ninety-five percent confidence intervals, P-values and the reversed transformed mean differences in original units were also reported. Three sets of multivariable regression analyses were then conducted; LV SF outcomes versus. (a) PDM or DM status controlling for main covariates (Table 3), (b) PDM or DM status controlling for main covariates with additional control for diabetes duration (Table 4) and (c) HbA1c categories (< 5.7%, 5.7- < 6 to < 6.5%, 6.5% to < 8.0% and ≥ 8.0%) controlling for main covariates, diabetes duration and carvedilol equivalent dose (Table 5). Analyses of BSA indexed outcomes were not further controlled for body habitus (BMI/BSA). Given the common co-occurrence of DM and hypertension and the potential joint effect on heart disease [31], the interaction of hypertension and either PDM or DM with LV SF outcomes on the multiplicative scale was examined (Figs. 2, 3). Supplementary analysis examined the distribution and means of study variables among study participants compared with excluded participants with CAD, arrhythmia, valvular heart disease and ESRD (Additional file 1: Table 1). All statistical tests were 2-sided at a significance level of α = 0.05 using SAS version 9.4 © (SAS Institute Inc., Cary, N.C.).

## Results

The characteristics of the study sample including outcomes, independent variables and covariates are presented in Table 1. Unadjusted and adjusted linear regression analyses results for each of the LV SF outcomes by PDM and DM status or HbA1c categories are presented in Tables 2, 3, 4, 5 and Figs. 2, 3. For all regression analyses, differences in original unit means and percentage differences when compared with the reference measures are reported.

**Table 1:** Sample characteristics

Variables	No diabetes/prediabetes	Prediabetes	Diabetes	^¥^Sig	*n*
	Frequency (%)	Frequency (%)	Frequency (%)		
*Female*					
Male	300 (31.02)	259 (36.43)	178 (32.01)	0.057	2234
Female	667 (68.98)	452 (36.57)	378 (67.99)		
*Highest level of education*					
Less than high school	91 (9.41)	108 (15.19)	134 (24.10)	< 0.001	2234
High school/GED		148 (20.82)	117 (21.04)		
Vocational school, trade school, or college	719 (74.35)	455(63.99)	305 (54.86)		
^€^*Physical activity index*					
Poor	366 (37.85)	350 (49.23)	317 (57.01)	< 0.001	2234
Intermediate	346 (37.64)	218 (30.66)	159 (28.60)		
Ideal	237 (24.51)	143 (21.11)	80 (14.39)		
*Alcohol consumption*					
None	490 (50.67)	411 (57.81)	384 (69.06)	< 0.001	2234
Moderate and Heavy/At risk	477 (49.33)	300 (42.19)	172 (30.94)		
^β^*Smoking status (AHA classification)*					
Current smoker	103 (10.65)	78 (10.97)	55 (9.89)	0.820	2234
Former smoker & Never smoker/Quit > 12 months	864 (89.35)	633 (89.03)	501 (90.11)		
^∞^*Nutrition Status*					
Poor	389 (40.23)	312 (43.88)	326 (58.63)	< 0.001	2234
Intermediate and Ideal Health	578 (59.77)	399 (56.12)	230 (41.37)		
Used crack or cocaine in any form	41 (4.24)	25 (3.52)	12 (2.16)	0.103	2234
Dyslipidemia	501 (51.81)	460 (64.70)	270 (48.56)	< 0.001	2234
^α^*CKD*					
No CKD	951 (98.35)	678 (95.36)	506 (91.01)	< 0.001	2234
CKD III & IV	16 (1.65)	33 (4.64)	50 (8.99)		
^¥^Hypertension	426 (44.36)	460 (64.70)	434 (78.06)	< 0.001	2234
Beta Blocker Medications	58 (6.00)	96 (13.50)	81 (14.57)	< 0.001	2234
Calcium Channel Blocker	141 (14.58)	155 (21.80))	151 (27.16)	< 0.001	2234
ACE/ARB Medications	187 (19.34)	182 (25.60)	301 (54.14)	< 0.001	2234
Diuretics	243 (25.13)	287 (40.37)	278 (50.00)	< 0.001	2234
Age in years	50.24 (12.27)	56.41 (10.61)	58.35 (11.28)	< 0.001	2234
Heart Rate (Beats/Minute)	66.03 (10.78)	65.44 (11.23)	69.89 (12.23)	< 0.001	2234
BMI (kg/m^2^)	30.63 (6.87)	32.85 (7.01)	34.49 (7.22)	< 0.001	2234
BSA (m^2^)	1.96 (0.23)	2.03 (0.22)	2.06 (0.23)	0.748	2234
HbA1c (%)	5.22 (0.33)	5.87 (0.30)	7.52 (1.70)	< 0.001	2234
Time since diabetes diagnosed in years (DM Duration)	0 (0)	0 (0)	10.12 (9.80)	N/A	2082
Left Ventricular Ejection Fraction %	61.66 (6.66)	62.44 (7.19)	62.28 (7.34)	0.057	2234
Left Ventricular Fractional Shortening %	39.28 (5.84)	39.31 (6.22)	39.98 (6.54)	0.174	1600
Left Ventricular End Diastolic Volume Index ml/m^2^	34.47 (11.32)	34.33 (12.77)	35.04 (15.51)	0.666	2234
Left Ventricular End Systolic Volume Index ml/m^2^	17.59 (5.46)	16.89 (6.03)	17.08 (7.55)	0.061	2234
Myocardial Contraction Fraction %	58.60 (17.76)	56.18 (22.13)	53.10 (12.04)	< 0.001	2234
Relative Wall Thickness (cm)	0.34 (0.06)	0.36 (0.07)	0.37 (0.07)	< 0.001	2234
Stroke Volume Index (ml/m^2^)	37.90 (7.42)	37.25 (7.74)	37.30 (8.14)	0.159	2234
Cardiac Index (L/min/m^2^)	2.41 (0.58)	2.35 (0.56)	2.58 (0.63)	< 0.001	2234
Left Ventricular Mass Index (g/m^2^)	68.89 (14.77)	71.74 (16.67)	75.30 (18.06)	< 0.001	2234
Beta Natriuretic Peptide (pg/mL)	12.27 (17.72)	12.93 (19.08)	15.00 (27.50)	0.029	1760
^≠^Carvedilol dose equivalent (mg)	1.42 (7.35)	3.27 (10.72)	2.11 (8.15)	0.019	2078

^¥^Chi square, fishers exact tests were utilized for categorial variables and one-way ANOVA when appropriate

^€^Defined according to American Heart Association's Life's Simple 7 criteria for minutes/week of moderate or vigorous physical activity. Poor physical activity: 0 min/week of leisure‐time moderate or vigorous physical activity. Intermediate physical activity: > 0 and < 150 min/week of leisure‐time moderate physical activity. > 150 min per week of moderate-intensity aerobic activity or 75 min per week of vigorous aerobic activity, or a combination of both. βCurrent Smoker, former smoker (quit < 12 months) and Never smoker/Quit ≥ 12 months. ∞Nutrition Status based on AHA categorization; Components (based on 2000‐kcal diet): (a) Fruits and vegetables: ≥ 4.5 cups/day, (b) Fish: > 3.5 oz, twice per week, (c) Sodium: < 1500 mg/day, (d) Sugary beverages: < 450 kcal/wk and (e) Whole grains: ≥ 3 servings/day. Poor Health: 0–1 components, Intermediate Health: 2–3 components and Ideal Health: 4–5 components. ¥Hypertension-BP > 140/90 mmHg and taking medications for HTN. αCKD-CKD stage II–III evidence by eGFR 60 to 89 mL/min per 1.73 m2 (MDRD method) or evidence of kidney damage urine albumin/creatinine ratio > 30 mg/dl. ≠ Carvedilol dose equivalent

**Table 2:** Left ventricular outcomes versus PDM/DM: Unadjusted Model (Reference = No PDM/DM)

Left Ventricular Measures	Prediabetes		Sig	Diabetes		Sig	*n*
	Mean difference	Percent parameter difference (95% CI)		Mean difference	Percent parameter difference (95% CI)		
Left Ventricular Ejection Fraction (%)	0.68	1.11 (− 0.07, 2.30)	0.006	0.50	0.80 (− 0.46, 2.09)	0.212	2234
Left Ventricular Fractional Shortening (%)	− 0.15	− 0.39 (− 2.33,1.60)	0.699	0.54	1.39 (− 0.77,3.60)	0.209	1600
Left Ventricular End Diastolic Volume (ml/m^2^)	− 1.50	− 2.78 (− 4.56, − 0.97)	0.003	− 1.38	− 2.56 (− 4.48, − 0.60)	0.11	2234
Left Ventricular End Systolic Volume (ml/m^2^)	− 0.77	− 4.74 (− 7.76, − 1.61)	0.003	− 0.84	− 5.14 (− 8.38, − 1.78)	0.003	2234
Stroke Volume Index (ml/m^2^)	− 0.75	− 2.03 (	0.046	− 0.77	− 2.09 (− 4.20, 0.06)	0.056	2234
Cardiac Index (L/min/m^2^)	− 0.01	− 2.75 (− 4.97, − 0.47)	0.018	0.15	6.48 (3.86, 9.16)	< 0.001	2234
Myocardial Contraction Fraction (%)	− 2.92	− 5.37 (− 7.43, − 3.26)	< 0.001	− 5.55	− 9.95 (− 12.06, − 7.78)	< 0.001	2234
Left Ventricular Mass Index (g/m^2^)	2.48	3.52 (1.29, 5.81)	0.002	5.98	8.72 (6.20, 11.31)	< 0.001	2234
Relative Wall Thickness (cm)	0.02	4.88 (3.10, 6.68)	< 0.001	0.03	8.10 (6.14, 10.11)	< 0.001	2234

**Table 3:** Left ventricular outcomes versus PDM/DM: Adjusted Model #1 (Reference = No PDM/DM)

Left Ventricular Measures	Prediabetes		Sig	Diabetes		Sig	*n*
	Mean difference	Percent parameter difference (95% CI)		Mean difference	Percent parameter difference (95% CI)		
Left Ventricular Ejection Fraction (%)	0.39	0.64 (− 0.59,1.88)	0.308	0.13	0.21 (− 1.20,1.64)	0.767	2,234
Left Ventricular Fractional Shortening (%)	− 0.61	− 1.57 (− 3.56,0.46)	0.494	− 0.08	0.22 (− 2.58,2.20)	0.859	1,600
Left Ventricular End Diastolic Volume (ml/m^2^)	− 1.43	− 2.65 (− 4.49, − 0.78)	0.006	− 1.44	− 2.66 (− 4.89, − 0.27)	0.015	2,234
Left Ventricular End Systolic Volume (ml/m^2^)	− 0.59	− 3.59 (− 6.73, − 0.34)	0.031	− 0.60	− 3.67 (7.23,—0.02)	0.051	2,234
Stroke Volume Index (ml/m^2^)	− 0.80	− 2.18 (− 4.21, − 0.12)	0.017	− 0.96	− 2.61 (− 4.89, − 0.27)	0.029	2,234
Cardiac Index (L/min/m^2^)	− 0.06	− 2.32 (− 4.62,0.03)	0.053	0.14	6.03 (3.20,8.93)	< 0.001	2,234
Myocardial Contraction Fraction (%)	− 0.40	− 0.74 (− 2.91,1.49)	0.513	− 1.90	− 3.43 (− 5.84, − 0.95)	0.007	2,234
Left Ventricular Mass Index (g/m^2^)	− 0.11	− 0.16 (− 1.99,2.33)	0.891	2.29	3.31 (0.81,5.89)	0.009	2,234
Relative Wall Thickness (cm)	0.01	1.45 (− 0.03,3.25)	0.107	0.01	3.13 (1.11, 5.25)	0.002	2,234

Each row represents a separate model for Left ventricular outcomes and were adjusted for Hypertension age, gender, physical activity, highest level of education, nutrition pattern, dyslipidemia smoking status, alcohol use, use of crack or cocaine, beta blocker, calcium channel blockers, diuretics, Angiotensin converting enzyme inhibitor, Angiotensin Receptor Blocker. Only LVEF, LV FS, MCF and RWT which were additionally controlled for log of BMI

**Table 4:** Left ventricular outcomes versus PDM/DM: Adjusted Model #2 (Reference = No PDM/DM)

Left Ventricular Measures	Prediabetes		Sig	Diabetes		Sig	*n*
	Mean difference	Percent parameter difference (95% CI)		Mean difference	Percent parameter difference (95% CI)		
Left Ventricular Ejection Fraction (%)	0.40	0.65 (− 0.58, 1.91)	0.302	− 0.14	− 0.233 (− 2.15, 1.72)	0.814	2082
Left Ventricular Fractional Shortening (%)	− 0.68	− 1.75 (− 3.73, 0.28)	0.090	− 0.16	− 0.42 (− 3.60, 2.86)	0.798	1512
Left Ventricular End Diastolic Volume (ml/m^2^)	− 1.15	− 2.82 (− 4.66, − 0.95)	0.003	− 0.66	− 1.23 (− 4.15, 1.77)	0.417	2082
Left Ventricular End Systolic Volume (ml/m^2^)	− 0.62	− 3.73 (− 6.88, − 0.47)	0.025	− 0.04	− 0.27 (− 5.35, 5.08)	0.920	2082
Stroke Volume Index (ml/m^2^)	− 0.87	− 2.36 (− 4.37, − 0.31)	0.024	− 0.71	− 1.92 (− 5.07, 1.33)	0.243	2082
Cardiac Index (L/min/m^2^)	− 0.05	− 2.26 (− 4.53, 0.07)	0.058	0.12	5.10 (1.28, 9.07)	0.009	2082
Myocardial Contraction Fraction (%)	− 0.04	− 0.81 (− 2.98, 1.40)	0.469	− 1.64	− 2.96 (− 6.25, 0.45)	0.089	2082
Left Ventricular Mass Index (g/m^2^)	< 0.01	0.01 (− 2.14, 2.21)	0.997	2.68	3.28 (− 0.19, 6.88)	0.064	2082
Relative Wall Thickness (cm)	0.01	1.54 (− 0.23, 3.35)	0.089	0.01	2.63 (− 0.16, 5.50)	0.065	2082

Each row represents a separate model for Left ventricular outcomes and were adjusted for diabetes duration, Hypertension age, gender, physical activity, highest level of education, nutrition pattern, dyslipidemia smoking status, alcohol use, use of crack or cocaine, beta blocker, calcium channel blockers, diuretics, Angiotensin converting enzyme inhibitor, Angiotensin Receptor Blocker. Only LVEF, LV FS, MCF and RWT which were additionally controlled for log of BMI

**Table 5:** Left ventricular outcomes versus HbA1c Categories: Adjusted Model. Reference HbA1c < 5.7%)

Left Ventricular Measures	HbA1c 5.7–< 6.5%			HbA1c 6.5–< 8.0%			HbA1c ≥ 8.0%			*n*
	Mean difference	Percent parameter difference (95% CI)	Sig	Mean difference	Percent parameter difference (95% CI)	Sig	Mean difference	Percent parameter difference (95% CI)	Sig	
Left Ventricular Ejection Fraction (%)	0.11	0.18 (− 1.00, 1.39)	0.763	− 1.34	− 2.18 (− 4.55, 0.25)	0.078	− 0.20	− 0.32 (− 2.69, 2.09)	0.787	2082
Left Ventricular Fractional Shortening (%)	− 0.39	− 1.02 (− 2.96, 0.97)	0.314	− 1.02	− 2.64 (− 6.63, 1.50)	0.208	− 0.51	− 1.34 (− 5.30, 2.79)	0.519	1512
Left Ventricular End Diastolic Volume (ml/m^2^)	− 1.19	− 2.25 (− 4.04, − 0.42)	0.016	− 1.45	− 2.75 (− 6.40, 1.05)	0.152	− 2.11	− 3.97 (− 7.47, − 0.34)	0.032	2082
Left Ventricular End Systolic Volume (ml/m^2^)	− 0.40	− 2.44 (− 5.53, 0.75)	0.133	− 0.20	1.25 (− 5.29, 8.23)	0.716	− 0.19	− 1.15 (− 7.34, 5.46)	0.726	2082
Stroke Volume Index (ml/m^2^)	− 0.75	− 2.13 (− 4.07, − 0.14)	0.036	− 1.74	− 4.84 (− 8.72, − 0.80)	0.020	− 2.09	− 5.81 (− 9.53, − 1.93)	0.004	2082
Cardiac Index (L/min/m^2^)	− 0.06	− 2.28 (− 4.49, − 0.02)	0.048	0.01	0.14 (− 4.49, 5.00)	0.952	0.11	4.85 (0.15, 9.78)	0.043	2082
Myocardial Contraction Fraction (%)	− 0.38	− 0.72 (− 2.80, 1.41)	0.507	− 1.19	− 2.21 (− 6.40, 2.15)	0.315	− 2.94	− 5.38 (− 9.32, − 1.26)	0.011	2082
Left Ventricular Mass Index (g/m^2^)	− 0.04	− 0.06 (− 2.14, 2.06)	0.953	− 0.71	− 1.01 (− 5.22, 3.39)	0.647	1.42	2.06 (− 2.15, 6.46)	0.341	2082
Relative Wall Thickness (cm)	< 0.01	1.16 (− 0.53, 2.90)	0.180	< 0.01	1.12 (− 2.36, 4.71)	0.533	0.01	3.73 (0.26, 7.32)	0.035	2082

Each row represents a separate model for Left ventricular outcomes and were adjusted for diabetes duration, Hypertension age, gender, physical activity, highest level of education, nutrition pattern, dyslipidemia smoking status, alcohol use, use of crack or cocaine, carvedilol dose equivalent, calcium channel blockers, diuretics, Angiotensin converting enzyme inhibitor, Angiotensin Receptor Blocker. Only LVEF, LV FS, MCF and RWT which were additionally controlled for log of BMI

### LV EF and LV FS

No statistically significant difference in LV EF of LV FS was observed by PDM and DM status (Tables 3 and 4).

### LVEDVI

In participants with PDM compared with those without PDM or DM, LVEDVI was 2.65% lower (*p* = 0.006) Table 3. This effect remained even after adjustment for DM duration (− 2.82% *p* = 0.003) Table 4. Participants with DM compared with the reference group had a 2.66% lower LVEDVI (*p* = 0.015) Table 3 though the effect was not observed after adjustment for DM duration (*p* = 0.417) Table 4.

### LVESVI

In participants with PDM compared with those without PDM or DM, LVESVI was 3.59% lower (*p* = 0.031) Table 3. This effect remained even after adjustment for DM duration (− 3.73%, *p* = 0.025) Table 4. No statistically significant difference in LVESVI was observed by DM status (Tables 3 and 4).

### SVI

In participants with PDM compared with those without PDM or DM, SVI was 2.18% lower (*p* = 0.017) Table 3. This effect remained even after adjustment for DM duration (− 2.36%, *p* = 0.024) Table 4. Participants with DM compared with the reference group had a 2.61% lower SVI (*p* = 0.029) Table 3 though the effect was not observed after adjustment for DM duration (*p* = 0.243) Table 4.

### CI

PDM was not associated with any difference in CI (Tables 3 and 4). In participants with DM compared with those without PDM or DM, CI was 6.03% higher (*p* < 0.001) Table 3. This effect remained even after adjustment for DM duration (5.10%, *p* = 0.009) Table 4.

### MCF

PDM was not associated with any difference in MCF (Tables 3 and 4). In participants with DM compared with those without PDM or DM, MCF was 3.43% lower (*p* = 0.007) Table 3. This effect was not observed after adjustment for DM duration (*p* = 0.089) Table 4.

### RWT

**Fig. 4:**
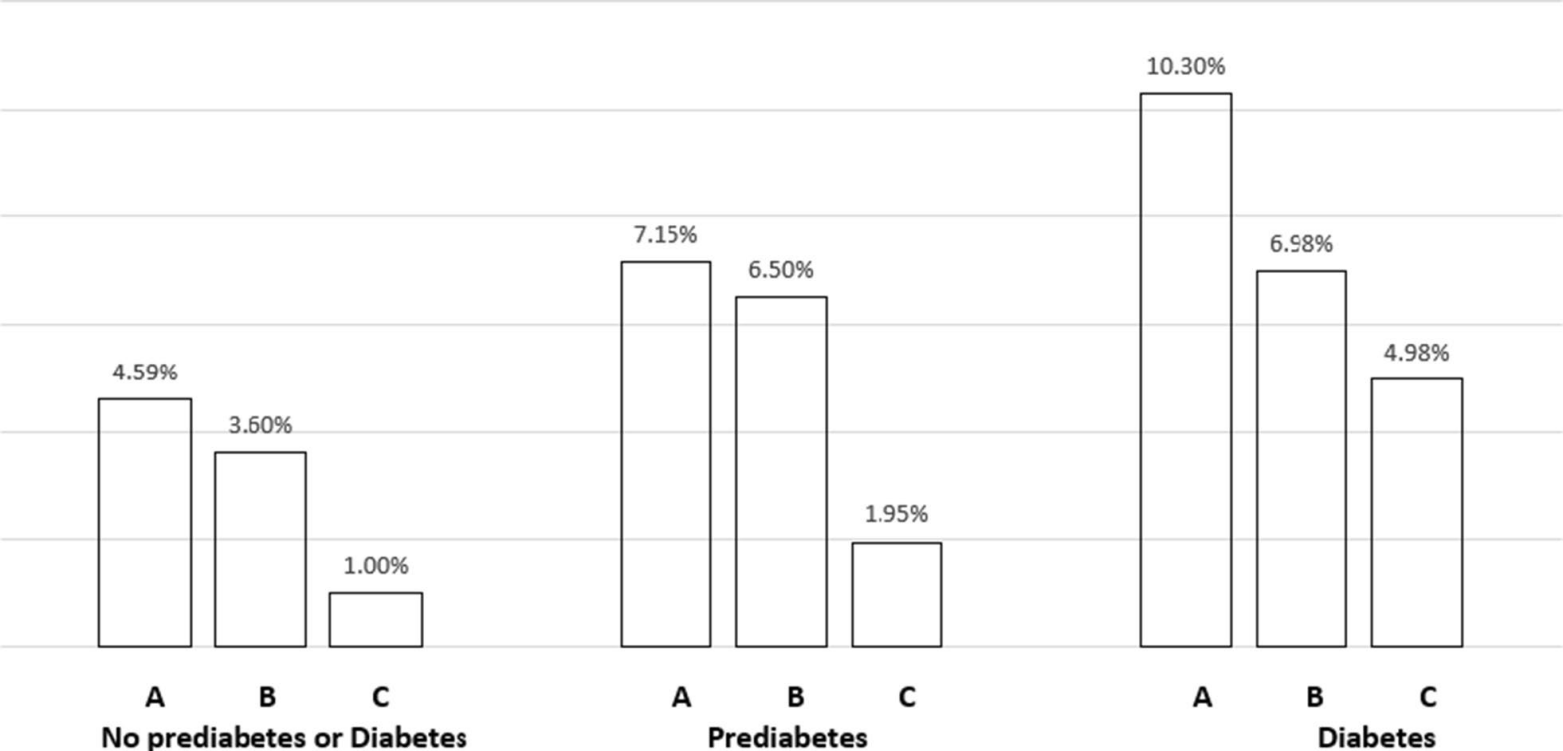
Left Ventricular Hypertrophy Pattern by PDM/DM Status. A = Eccentric Hypertrophy. B = Concentric Remodeling. C = Concentric Hypertrophy. P =  < 0.001

PDM was not associated with any difference in RWT (Tables 3 and 4). In participants with DM compared with those without PDM or DM, RWT was 3.13% higher (*p* = 0.002) Table 3. This effect was not observed after adjustment for DM duration (*p* = 0.065) Table 4. The distribution of left ventricular hypertrophy patterns by PDM and DM status is presented in Fig. 4. Eccentric hypertrophy was the predominant LVH pattern occurring with higher frequency among participants with PDM and DM when compared to those without either condition.

### LVMI

PDM was not associated with a difference in LVMI (Table 3) In participants with DM compared with those without PDM or DM, LVMI was 3.31% higher (*p* = 0.009) Table 3. This effect was not observed after adjustment for DM duration (*p* = 0.065) Table 4.

### Stratified analyses by HBA1c (Table 5)

In a subset of the study sample with available DM duration and carvedilol equivalent dose, we examined the relationship between categories of HbA1c and LV SF outcomes. These models controlled for the same similar covariates in Tables 3 and 4 in addition to duration of DM and carvedilol dose equivalent. Results showed that participants with HbA1c of ≥ 8% compared with HbA1c of < 5.7% had; 3.97% lower LVEDVI (*p* = 0.032), 5.81% lower SVI (*p* = 0.004), 4.85% higher CI (P = 0.043), 5.38% lower MCF (*p* = 0.011) and 3.73% higher RWT (*p* = 0.035). SVI was the only outcome measure that was significantly lower for participants with HbA1c of 6.5 to < 8.0% when compared the reference group (4.84% lower, *p* = 0.036). Participants with HbA1c of 5.7% to < 6.5% compared with the reference group had; a 2.25% lower LVEDVI (*p* = 0.016), 2.13% lower SVI (*p* = 0.036) and 2.28% lower CI (*p* = 0.048).

### Interaction of DM or PDM with hypertension on left ventricular structure and outcomes measures

Figures 2 and 3 presents results of several multivariable regression analyses exploring the multiplicative interaction of HTN on PDM and DM (respectively) on left ventricular structure and function outcomes. Results showed no statistically significant interaction on the multiplicative scale.

### Additional file 1: Table 1

Compares the distribution of study outcome variables and covariates by apriori excluded participants with CAD, arrythmia, valvular heart disease and ESRD status compared with selected participants without these conditions.

## Discussion

This study represents an examination of left ventricular structure and function among a population of African Americans who have DM without concurrent CAD, arrhythmia, valvular heart disease or ESRD. Similar studies are sparse in this population. Study findings show significantly lower LVEDVI, SVI, MCF but higher CI, RWT and LVMI among African Americans with DM compared with those without DM. These finding appear DM duration dependent except for CI which remained higher among individuals with DM compared to those without DM when DM duration was considered. PDM was associated with lower LVEDVI, LVESVI and SVI among compared with no PDM. We also observed important differences in the cardiac structure and function measures by DM control using HbA1c. Sensitivity analysis failed to show that interaction of DM with hypertension modified observed effects significantly. Study findings are further discussed below.

### Left ventricular end diastolic and systolic volumes and Stroke volume index

SVI is essentially the difference between the LVEDVI and LVESVI and has been strongly associated with all-cause mortality and adverse cardiac events [32, 33]. While the pathophysiology of cardiomyopathy and structural heart changes in DM has not been fully elucidated, some evidence suggests that left ventricular function and contractility is impaired early in the course of the disease [34]. In our study, PDM but not DM was associated with decreased LVEDVI and LVESVI. SVI was lower among participants with DM compared with those without PDM/DM, though appeared confounded by DM duration. In contrast, SVI was lower among participants with PDM compared with those without PDM/DM. While temporal relationships are difficult to ascertain from cross sectional studies, these findings may suggest that changes in SVI occur early in the PDM to DM spectrum among African Americans. It remains unclear if the decrease in SVI results in adaptive physiologic changes in participants with DM but not PDM. The relationship between HbA1c and SVI however showed a clearer relationship between SVI and glycemic status. Specifically, SVI progressively decreased as HbA1c increased in comparison to the normal HbA1c (< 5.7%). LVEDVI also similarly decreased with increasing HbA1c. These findings should however be interpreted with caution as HbA1c is dynamic and may not represent long term glycemic status.

This study’s results compare with prior studies including Jensen et al. UK biobank cardiovascular magnetic resonance sub-study that found a decrease in SVI among participants with DM and Bertoni et al. study that found a decrease in stroke volume among African Americans with DM with prior Cardiovascular disease [35]^36^. Both studies did not however account for DM duration or HbA1c in their models like we did in this study. The pathophysiologic basis for the findings in our study is supported by prior evidence. Specifically, metabolic abnormalities (affecting glucose and free fatty acid), abnormal calcium homeostasis, myocardial apoptosis, myocardial fibrosis, small vessel ischemia and microangiopathy theoretically result in myocardial stiffness, impaired relaxation and lower end diastolic volumes [11, 37–42]. Our study suggests that LVEDVI and SVI may be a clinically relevant measure to consider in African Americans with abnormal glycemic status though further longitudinal studies are required to more clearly elucidate these relationships.

### Cardiac Index

In our study, CI was higher for participants with DM compared to those without PDM/DM and similarly higher for participants with HbA1c ≥ 8.0% compared with normal HbA1c (< 5.7%). Paradoxically, HbA1c of 5.7 to < 6.5% compared with normal HbA1c was associated with lower CI. Study finding of higher CI are similar though not of the same magnitude as findings in the Strong Heart Study [43]. Specifically, this study’s observed mean CI difference was lower though the Strong Heart Study was of Native American not African Americans and did not control for similar covariates including DM duration as done in this study.

CI is typically the product of SV and heart rate at time of volumetric assessment. As discussed in the previous section, lower SVI and LVEDVI among individuals with DM or poor control (higher HbA1c) may reflect impaired ventricular filling secondary to relaxation impairments or decreased filling time [11, 37]. Our observed higher CI for DM and HbA1c ≥ 8.0% is thus likely attributable to a higher resting heart rate [44]. The higher resting heart rate among individuals with DM is often attributed to cardiac autonomic neuropathy (CAN) which is increasingly recognized as an important physiologic change among individuals with DM resulting from early cardiac parasympathetic denervation [11, 45]. Ewing and Balcıoğlu suggest that among individuals with DM, vagal denervation results a dominant sympathetic tone and resting tachycardia. They reported that while tachycardia eventually diminishes secondary to progressive sympathetic nerve fiber damage, increased resting heart rate persists among individuals with DM [46, 47]. CAN may also affect myocardial blood flow in denervated neuropathic individuals with DM when compared with non -neuropathic patients with DM [48]. In our study, we observed a progressive increase in CI as HbA1c increased that may give an insight to the glycemic control range at which CAN effect on CI occurs. This observed relationship should be interpreted with caution as HbA1c is dynamic and may not always represent long-term glycemic status. While we did not explicitly study CAN, further studies to elucidate the role of heart rate on CI among African Americans with DM may be relevant. Furthermore, elevated CI is likely not sustainable as clinical cardiomyopathy and HF eventually may occur in individuals with DM. The exact progression to HF and pathogenesis of these changes cannot however be elucidated from this study and requires further prospective studies.

### Myocardial contraction fraction

MCF is a unitless three-dimensional volumetric measure of myocardial shortening proposed by King et al. as potentially outperforming traditional shortening measures like LV EF [24]. Several studies have since demonstrated a strong association between MCF and adverse cardiovascular outcomes [49, 50]. MCF is essentially a ratio of stroke volume to left ventricular volume and is independent of chamber size and geometry. In fact, King et al. hypothesized that MCF is perhaps a more useful measure of myocardial function in part because its derivation lacks the influence of LVEDV on ventricular shortening. This is of importance in our study because we sought to evaluate the independent effect of glycemic status on MCF as an LV structure and function without the influence of underlying cardiac chamber changes.

In this study, we observed that MCF was lower for participants with DM compared to those without PDM/DM. In the model controlled for DM duration however, we found no clear association of DM with MCF. In contrast, HbA1c ≥ 8.0% compared with the reference HbA1c < 5.7% was associated with a lower MCF. Glycemic control may thus be a more important determinant of MCF. Few studies have evaluated the relationship of glycemic status on MCF among African Americans as explored in this study. Abdalla et al. using the Multi-Ethnic Study of Atherosclerosis (MESA) did demonstrate that DM was associated with the lower MCF, though utilized cardiac magnetic resonance imaging (cMRI) rather than echocardiography [51]. The findings of this study may inform further studies regarding glycemic status and MCF among African Americans.

### Left ventricular mass index and relative wall thickness

No statistically significant relationship between DM or HbA1c and LVMI was obsereved after we controlled for DM duration. While larger LVMI has been among individuals with DM compared with no DM further confering poorer cardiovascular outcomes [36, 43, 52, 53], few studies have examined this relationship among African Americans without CAD, arrhythmia, valvular heart disease or ESRD. The LVMI finding in this study is in contrast to these prior studies and should be interpreted with caution as LVMI is generally higher for African Americans and detection of large differences in a homogenous sample may be difficult [54].

In our study, RWT was higher among participants with HbA1c ≥ 8.0% compared with HbA1c < 5.7% though was not significantly different for DM versus no PDM/DM participants. Glycemic control may thus be an important determinant of RWT. There is mixed evidence regarding the influence of co-occurring hypertension and DM on left ventricular hypertrophy with some studies reporting that it is hypertension dependent others maintain that it is hypertension independent [55–57]. In our study the interaction between PDM or DM with hypertension on both RWT and LVMI was not statistically significant. Analyses presented in Fig. 4 shows that eccentric remodeling was the predominant hypertrophy pattern for individuals with PDM and DM. This finding may underly the independent influence of DM on hypertrophy patterns though both concentric and eccentric hypertrophy patterns may can occur in normotensive individuals with DM [58, 59].

### Left ventricular ejection fraction and left ventricular fractional shortening

In our study, no statistically significant difference in LV EF or LV FS was observed for PDM, DM or HbA1c. This finding is not inconsistent with emerging evidence despite evidence to the contrary [60]. The sensitivity of LV EF for detecting early systolic dysfunction may in fact be poor despite its wide clinical use [61]. Nakai el al for example demonstrated that 2D speckle tracking echocardiography (STE) evaluation of longitudinal strain showed evidence of subclinical LV longitudinal dysfunction preferentially and frequently in asymptomatic DM patients with normal LV EF [14]. Further studies to evaluate the relationship between more sensitive measures of left ventricular dysfunction with DM may elucidate these relationships more appropriately. STE data was not evaluated in JHS.

## Conclusion

From a clinical perspective, study findings highlight important abnormalities in LV structure and function among individuals with abnormal glycemic status that may present intervention opportunities though further causal studies are required. While the magnitude of observed differences may not be of overt clinical utility, they should be considered in context of the cross-sectional design of the study and the potential for observing larger effect sizes in longitudinal studies. Many of the LV structure and function parameters that we examined were DM duration and HbA1c level dependent further highlighting the need for early case finding and DM control. The strong association of PDM with SVI and DM or HbA1c with CI requires further pathophysiologic enquiry not addressed in our study. Specifically, is there a temporal relationship between glycemic status, CI, stroke volume and heart rate?. Is heart rate perhaps an important prognostic marker for subclinical LV changes and ultimately clinical HF? Do changes in LVEDVI occur much earlier than thought particularly even among individuals with PDM? Study findings also suggest a potential prognostic value of MCF among individuals with uncontrolled DM especially given the potential limitations of LV EF as a screening tool for subclinical and early systolic changes. Overall, this study in our impression contributes to the limited literature regarding LV structure and function in African Americans who are at considerable risk for DM associated structural cardiac changes.

### Study limitations

This study is subject to important limitations. First, the cross-sectional design limits the ability to make clear inference regarding causality. The findings however suggest potential causal hypotheses in support of further studies. Despite efforts to exclude participants with silent ischemia using for example EKG evidence of prior ischemia, some potential for missing underlying ischemia still exists. Data regarding DM duration and beta blocker use was not available for all participants resulting in smaller subset analytic samples.DM duration is subject to potential recall bias and systematic error. The lack of tissue doppler studies in JHS limited our ability to evaluate diastolic parameters. Finally, limited M-mode LV FS outcome data may affect the power study observations.

## Supplementary Information

**Additional file 1**.** Supplementary Table 1**. Distribution of study variables by exclusion criteria (presence or absence of CAD, arrhythmia, valvular Disease or ESRD).

## Data Availability

The data that support the findings of this study are available from [Jackson Heart Study] but restrictions apply to the availability of these data, which were used under license for the current study, and so are not publicly available. Data are however available from the authors upon reasonable request and with permission of [Jackson Heart Study].

## Abbreviations

DM: Diabetes Mellitus
CAD: Coronary artery disease
ESRD: End-stage renal disease
PDM: Prediabetes
JHS: Jackson Heart Stud
LV EF: Left ventricular ejection fraction
LV FS: Left ventricular fractional shortening
SVI: Stroke volume index
CI: Cardiac Index
LVEDVI: Left ventricular end diastolic volume index
LVESVI: Left ventricular end systolic volume index
RWT: Relative wall thickness
MCF: Myocardial contraction fraction
LVMI: Left ventricular mass index
D-CM: Diabetic cardiomyopathy
LV: Left ventricular
LV SF: Left ventricular structure and function
LVEDD: Left Ventricular End Diastolic Diameter
LVESD: Left Ventricular End Systolic Diameter
LVM: Left Ventricular Mass
HbA1c: Glycated Hemoglobin
LDL-C: Low density lipoproteins
HDL-C: High density lipoproteins
CKD: Chronic Kidney Disease
FFQ: Food Frequency Questionnaire
MDRD: Modification of Diet in Renal Disease
AHA: American Heart Association
MSRA: Medication Survey Form
ACE: Angiotensin converting enzyme inhibitor
ARB: Angiotensin receptor blockers
BSA: Body surface area
BMI: Body mass index
