# Supplementary material for: Glycemic status, non-traditional risk and left ventricular structure and function in the Jackson Heart Study
Source: BMC Cardiovasc Disord. 2022 Apr 21;22:186. doi: 10.1186/s12872-022-02605-w (PMC9022283; doi:10.1186/s12872-022-02605-w)
Supplement: Supplementary file 1 — Additional file 1. Supplementary Table 1. Distribution of study variables by exclusion criteria (presence or absence of CAD, arrhythmia, valvular Disease or ESRD). [file 12872_2022_2605_MOESM1_ESM.docx]

**Supplementary Table 1:** Distribution of study variables by exclusion criteria (presence or absence of CAD, Arrhythmia, Valvular or ESRD *n*=4052

| **Variables** | **No CAD, Arrhythmia, Valvular or ESRD** | | **CAD, Arrhythmia, Valvular or ESRD** | | **Sig.** |
| --- | --- | --- | --- | --- | --- |
|  | ***Frequency* (%)** | ***n*** | ***Frequency* (%)** | ***n*** |  |
| Female   - Male - Female | 1044(36.88%)  1787(63.12%) | 2831 | 413(33.82%)  808(66.18%) | 1221 | 0.063 |
| Highest level of education   - Less than high school - High school/GED - Vocational school, trade school, or college | 402(14.20%)  546(19.29%)  1883(66.51%) | 2831 | 285(23.34%)  267(21.87%)  669(54.79%) | 1221 | <0.001 |
| Physical activity index   - Poor - Intermediate - Ideal | 1300(45.92%)  941(33.24%)  590(16.17%) | 2831 | 649(53.15%)  368(30.14%)  204(16.71%) | 1221 | <0.001 |
| Alcohol Consumption   - None - Moderate or Heavy/At risk | 1555(56.93%)  1276(45.07%) | 2831 | 822(67.32%)  399(32.68%) | 1221 | <0.001 |
| ^β^Smoking status (AHA classification)   - Current smoker - Never smoker/ Former smoker | 330(11.66%)  2501(88.34%) | 2831 | 152(12.45%)  1069(87.55%) | 1221 | 0.475 |
| ^∞^Nutrition Status   - Poor - Intermediate and Ideal Health | 1186(41.89%)  1645(58.11%) | 2831 | 575(47.09%)  646(52.91%) | 1221 | 0.002 |
| Used crack or cocaine | 122 (4.31%) | 2831 | 33(2.70%) | 1221 | 0.014 |
| Dyslipidemia   - No - Yes | 1106(39.07%)  1725(60.93%) | 2831 | 455(37.26%)  766(62.74%) | 1221 | 0.003 |
| CKD   - No CKD - CKD III & IV | 2728(93.36%)  103(3.64%) | 2831 | 1088(89.11%)  133(10.89%) | 1542 | <0.001 |
| Hypertension   - No - Yes | 1403(49.56%)  1428(50.44%) | 2831 | 368(30.14%)  853(69.86%) | 1221 | <0.001 |
| Left Ventricular Hypertrophy   - None - Concentric hypertrophy - Concentric remodeling - Eccentric Hypertrophy | 2433(85.94%)  188(6.64%)  149(5.26%)  61(2.15%) | 2831 | 956(78.30%)  162(13.27%)  59(4.83%)  44(3.60%) | 1221 | <0.001 |
| Diabetes Mellitus (Type I & I) or Prediabetes   - No - Prediabetes - Diabetes | 1306(46.14%)  923(32.60%)  602(21.26%) | 2831 | 462(37.84%)  432(35.38%)  327(26.78%) | 1221 | <0.001 |
| Beta Blocker Medications | 235(10.52%) | 2831 | 276(25.34%) | 1089 | <0.001 |
| Calcium Channel Blocker | 447(20.01%) | 2831 | 306(28.10%) | 1089 | <0.001 |
| ACE/ARB Medications | 670(29.99%) | 2831 | 442(40.59%) | 1089 | <0.001 |
| Diuretics | 808(36.17%) | 2831 | 488(44.81%) | 1089 | <0.001 |
| **Variables** |  |  |  |  |  |
|  | **Mean ± Std** | ***n*** | **Mean ± Std** | ***n*** | **Sig.** |
| Age in years | 52.51 ± 12.42 | 2831 | 59.81(11.63) | 1221 | <0.001 |
| BMI | 32.04 ±7.32 | 2831 | 31.40 ±7.01 | 1221 | 0.010 |
| Left Ventricular Ejection Fraction (%) | 63.24 (7.59) | 2831 | 62.44 (9.55) | 1221 | 0.010 |
| Left Ventricular Fractional Shortening % | 39.20 (6.06) | 2091 | 39.06 (7.29) | 842 | 0.618 |
| Left Ventricular End Diastolic Volume Index ml/m^2^ | 54.85 (10.39) | 2831 | 58.09 (14.76) | 1221 | <0.001 |
| Left Ventricular End Systolic Volume Index ml/m^2^ | 34.88 (12.88) | 2831 | 38.25 (22.45) | 1221 | <0.001 |
| Myocardial Contraction Fraction % | 56.62 (15.25) | 2831 | 54.30 (12.65) | 1221 | <0.001 |
| Stroke Volume Index (ml/m^2^) | 37.47 (7.63) | 2831 | 38.64 (8.60) | 1221 | <0.001 |
| Cardiac Index (L/min/m^2^) | 2.42 (0.58) | 2831 | 2.43 (0.61) | 1221 | 0.378 |
| Relative Wall Thickness cm | 0.35 (0.06) | 2831 | 0.037 | 1221 | 0.074 |
| Left Ventricular Mass Index (g/m^2^) | 71.00 (16.52) | 2831 | 77.79 (32.10) | 1221 | <0.001 |

^¥Chi square was utilized for categorial variables and one-way ANOVA when appropriate.^

^≠ Arrhythmia includes Atrial flutter, Atrial fibrillations, Major ventricular tachyarrhythmia’s € Defined according to American Heart Association's Life's Simple 7 criteria for minutes/week of moderate or vigorous physical activity. Poor physical activity: 0 minutes/week of leisure‐time moderate or vigorous physical activity. Intermediate physical activity: >0 and <150 minutes/week of leisure‐time moderate physical activity. >150 minutes per week of moderate-intensity aerobic activity or 75 minutes per week of vigorous aerobic activity, or a combination of both. βCurrent Smoker, former smoker (quit < 12 months) and Never smoker/Quit ≥ 12 months. ∞Nutrition Status based on AHA categorization; Components (based on 2000‐kcal diet):a)Fruits and vegetables: ≥ 4.5 cups/day, b)Fish: > 3.5 ounces, twice per week, c) Sodium: < 1500 mg/day, d)Sugary beverages: < 450 kcal/wk and e) Whole grains: ≥ 3 servings/day. Poor Health: 0-1 components, Intermediate Health: 2-3 components and Ideal Health: 4-5 components. ¥Hypertension-BP>140/90mmHg and taking medications for HTN. αCKD- CKD stage II-III evidence by eGFR 60 to 89 mL/min per 1.73 m2 (MDRD method) or evidence of kidney damage urine albumin/creatinine ratio >30mg/dl^
